# Variable Gene Copy Number in Cancer-Related Pathways Is Associated With Cancer Prevalence Across Mammals

**DOI:** 10.1093/molbev/msaf056

**Published:** 2025-03-20

**Authors:** Sophie Matthews, Vahid Nikoonejad Fard, Marc Tollis, Cathal Seoighe

**Affiliations:** School of Mathematical and Statistical Science, University of Galway, Galway, Ireland; The SFI Centre for Research Training in Genomics Data Science, University of Galway, Galway, Ireland; School of Informatics, Computing and Cyber Systems, Northern Arizona University, Flagstaff, AZ, USA; School of Informatics, Computing and Cyber Systems, Northern Arizona University, Flagstaff, AZ, USA; Arizona Cancer Evolution Center, Arizona State University, Tempe, AZ, USA; School of Mathematical and Statistical Science, University of Galway, Galway, Ireland; The SFI Centre for Research Training in Genomics Data Science, University of Galway, Galway, Ireland

**Keywords:** gene copy number, comparative oncology, mammals, gene duplication, cancer, Peto's paradox

## Abstract

Cancer is a disease of multicellularity, observed across the tree of life. In principle, animals with larger body sizes and longer lifespans should be at increased risk of developing cancer. However, there is no strong relationship between these traits and cancer across mammals. Previous studies have proposed that increased copy number of cancer-related genes may enhance the robustness of cancer suppression pathways in long-lived mammals, but these studies have not extended beyond known cancer-related genes. In this study, we conducted a phylogenetic generalized least squares analysis to test for associations between copy number of all protein-coding genes and longevity, body size, and cancer prevalence across 94 species of mammals. In addition to investigating the copy number of individual genes, we tested sets of related genes for a relationship between the aggregated gene copy number of the set and these traits. We did not find strong evidence to support the hypothesis that adaptive changes in gene copy number contribute to the lack of correlation between cancer prevalence and body size or lifespan. However, we found several biological processes where aggregate copy number was associated with malignancy rate. The strongest association was for the gene set relating to transforming growth factor beta, a cytokine that plays a role in cancer progression. Overall, this study provides a comprehensive evaluation of the role of gene copy number in adaptation to body size and lifespan and sheds light on the contribution of gene copy number to variation in cancer prevalence across mammals.

## Introduction

The evolution of large body sizes and extended longevity is constrained by an increased risk of developing cancer (Erten and Kokko 2021). While the probability that any given cell will acquire mutations or other changes that cause it to undergo cancer transformation is typically very low, naively the probability that some cell in an organism initiates that the formation of a tumor should be higher in species with a larger number of cells (Caulin and Maley 2011). Similarly, species with extended lifespans have more time to accumulate oncogenic mutations, and this should lead to a higher lifetime risk of developing cancer (Caulin and Maley 2011). However, the relationship between body size, longevity, and cancer occurrence remains unclear (Vincze et al. 2021). Within species, the risk of cancer has been shown to increase with lifespan and size (Fleming, Creevy and Promislow 2011; White et al. 2014; Nunney 2018). In humans, cancer is considered an age-related disease, given that the incidence of most cancers increases with age (White et al. 2014). Height has also been correlated with cancer incidence in humans (Nunney 2018). However, large studies of pathological records suggest that cancer incidence among mammals is not directly related to either longevity or body size (Vincze et al. 2021). This observation is termed Peto's paradox (Peto et al. 1975).

Several hypotheses have been proposed to explain Peto's paradox, such as increased immune system efficiency (Koebel et al. 2007; Klein 2009), lower somatic mutation rates (Seluanov et al. 2018), differences in tissue architecture (Caulin and Maley 2011), and the duplication of tumor suppressor genes (García-Cao et al. 2002; Matheu et al. 2004). The duplication of tumor suppressor genes as a cancer prevention mechanism has been supported by evidence across mammalian species. For example, Sulak et al. demonstrated that the increase in TP53 copies in the Proboscidean lineage tracks the evolution of large body size (Sulak et al. 2016). TP53 is a tumor suppressor gene responsible for apoptosis, senescence, and cell cycle arrest in the presence of damaged DNA (Kumari et al., 2014). Additional gene copies have the potential to increase redundancy and, therefore, to confer robustness against inactivating mutations that may lead to a malignant phenotype (Leroi et al. 2003; Caulin and Maley 2011). As many as 20 copies of TP53 have been reported in the elephant genome (Abegglen et al. 2015; Caulin et al. 2015; Sulak et al. 2016), suggesting that the robustness conferred by additional copies of tumor suppressor genes could contribute to the decoupling of cancer risk from body size and longevity. However, increased copy number of TP53 has not been observed in other large, long-lived mammals, suggesting that expansion of this specific tumor suppressor gene is not a universal mechanism to reduce cancer risk in these species.

In a study investigating the copy number of all tumor suppressor genes, the naked mole rat was found to contain the largest number of tumor suppressor gene duplications of 63 queried mammalian species (Tollis et al. 2020). This finding is consistent with the extremely low rate of cancer in the species, which suggests that it has evolved effective mechanisms to prevent cancer. It has been suggested that the duplication of tumor suppressor genes may enhance the robustness of cancer suppression pathways, contributing to the low cancer incidence observed in the species. In both this example, and in the case of TP53, the reported number of copies includes pseudogenes, and many of the additional gene copies do not encode proteins. These genes are frequently the products of historical transposition events, and because they do not produce functional protein, they are unlikely to contribute to cancer suppression directly by conferring redundancy with respect to the function of the protein encoded by the tumor suppressor genes (Nunney 2022). Previous studies have also focused solely on the duplication of cancer-related genes and have not considered other genes. The classification of cancer-related genes is reliant on human gene annotation, and there is a lack of experimental evidence that human cancer genes share the same function in other species; thus, these studies may be excluding genes that have a role in preventing cancer in other species.

In this study, we estimated the copy number of all protein-coding genes in the genomes of 94 species from across the mammalian radiation. We applied the phylogenetic generalized least squares (PGLS) method to explore the relationship between gene copy number and longevity, body size, and cancer prevalence. In addition to this analysis, we used a novel gene set approach, applying PGLS to the aggregate gene copy number in functionally related sets of genes. This method revealed examples of gene sets in which the aggregate copy number is associated with changes in malignancy rate across the mammalian phylogenetic tree. Interestingly, our PGLS analysis did not find gene copy number to be associated with either longevity or body size, contrary to the hypothesis that adaptation to these life history traits is mediated by changes in gene copy number. As an alternative approach to the analysis of functionally related sets of genes, we also applied standard gene set analysis methods such as gene set enrichment analysis (GSEA) and overrepresentation analysis (ORA) to the results of the PGLS analysis of individual protein-coding genes. Overall, this study presents evidence that gene copy number contributes to the variation in malignancy rate across mammals, shedding light on the evolution of cancer prevalence.

## Results

### PGLS Analysis of Gene Copy Number and Life History Traits

**Fig. 1.**
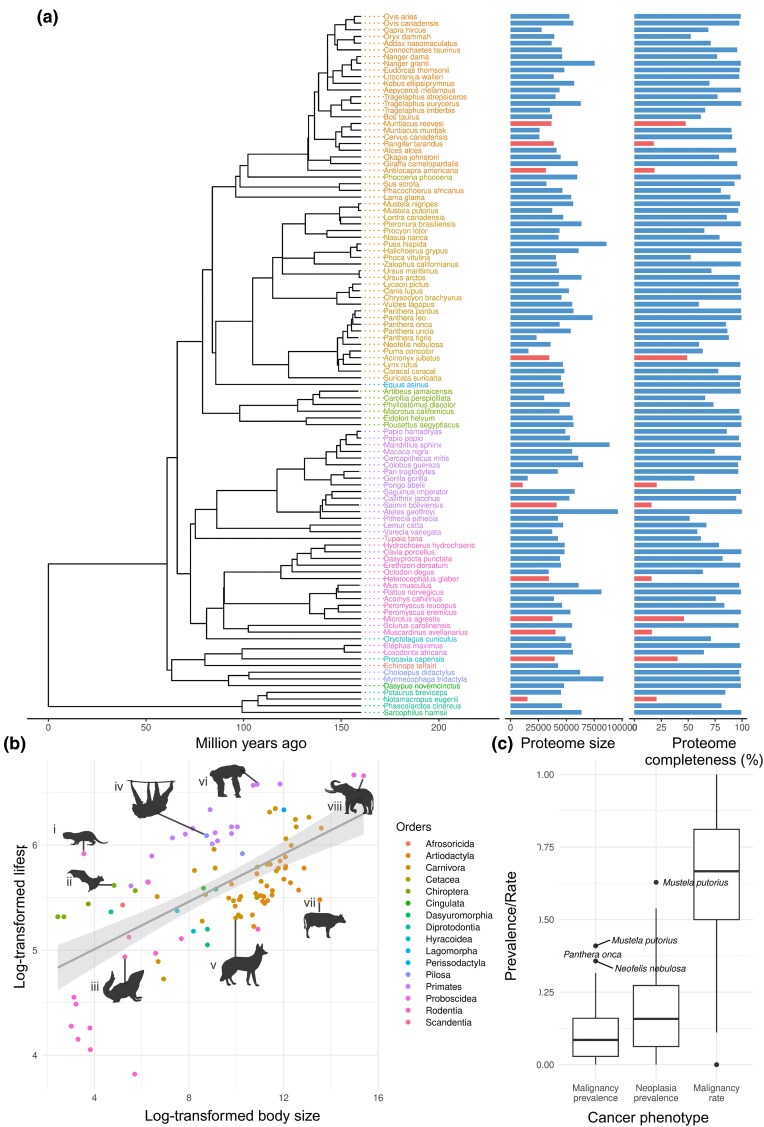
a) Phylogeny of the 105 mammalian genomes obtained for this study, inferred from timetree.org (Kumar et al. 2022). Colors represent mammalian orders. Bar graphs show the proteome size and completeness for each species. Red bars indicate proteomes with a completeness <50%, which were discarded from further analysis. b) Relationship between body size (g) and lifespan (months) for mammalian species used in this study. Gray line represents linear regression between log-transformed body size and log-transformed lifespan. Silhouettes from PhyloPic.org represent a range of mammalian orders included in this study (i) naked mole rat (*Heterocephalus glaber*, by Kai R. Caspar, https://creativecommons.org/licenses/by/3.0/), (ii) Egyptian fruit bat (*Rousettus aegyptiacus*, by Melissa Ingala, https://creativecommons.org/licenses/by/3.0/), (iii) large tree shrew (*Tupaia tana*), (iv) Linnaeus's two-toed sloth (*Choloepus didactylus*, by Kai R. Caspar, https://creativecommons.org/licenses/by/4.0/), (v) maned wolf (*Chrysocyon brachyurus*), (vi) chimpanzee (*Pan troglodytes*, by Kai R. Caspar, https://creativecommons.org/licenses/by/3.0/), (vii) cattle (*Bos taurus*) and (viii) African bush elephant (*Loxodonta africana*). c) Box plot shows the distribution of neoplasm phenotypes across the species studied.

We surveyed a total of 94 mammals in this study, encompassing species from all super orders of mammals (Afrotheria, Xenarthra, Euarchontoglires, and Laurasiatheria) (*Fig. 1*; supplementary data S1, Supplementary Material online). To estimate the copy number of genes in these species, we employed OrthoFinder to identify orthogroups encompassing protein-coding genes. A total of 29,648 orthogroups were identified. PGLS analysis did not reveal any association between gene copy number and life history traits: longevity, body size, neoplasia prevalence, or malignancy prevalence (false discovery rate [FDR]–adjusted *P*-value <0.05) (supplementary data S2, Supplementary Material online). However, we did find 15 orthogroups to be associated with malignancy rate (supplementary fig. S1 and table S1, Supplementary Material online). Interestingly, in all cases, the expansion of these orthogroups was correlated with a decrease in malignancy rate across the phylogenetic tree.

**Fig. 2.**
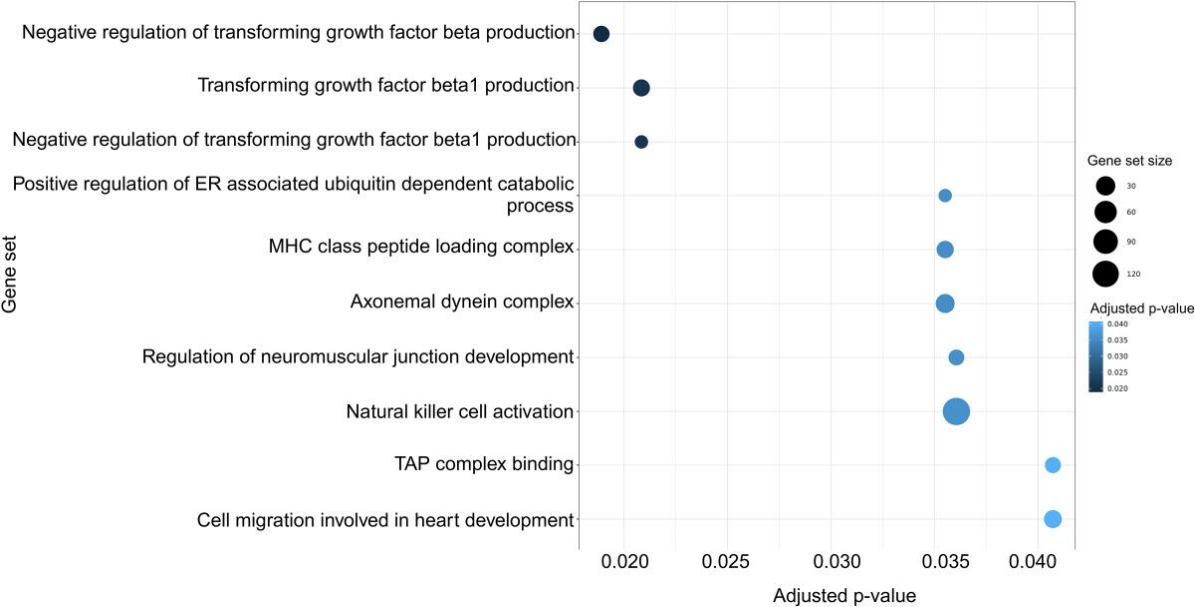
Dot plot shows gene sets with association (adjusted *P*-value <0.05) between aggregate gene count and malignancy rate according to PGLS models. The *x*-axis and color represent the significance of association (after correction for multiple testing using the Benjamini–Hochberg method). Gene sets are shown on the *y*-axis. The size of the dots represents the number of genes in each set.

We also tested for associations between the expansion and contraction of gene sets, using the aggregate gene count of genes in each set (supplementary data S3, Supplementary Material online). An increase in the aggregate count of one gene set (the biological process: “Keratinization”) was associated with decreased neoplasia prevalence, and 10 gene sets were associated with malignancy rate (adjusted *P*-value <0.05; Fig. 2; supplementary fig. S2, Supplementary Material online). Of the ten gene sets associated with malignancy rate, seven are categorized as biological processes, two cellular components, and one molecular function. Eight of these ten gene sets had a negative association between total gene count and malignancy rate, with higher aggregate gene counts associated with a lower rate of malignancy.

**Fig. 3.**
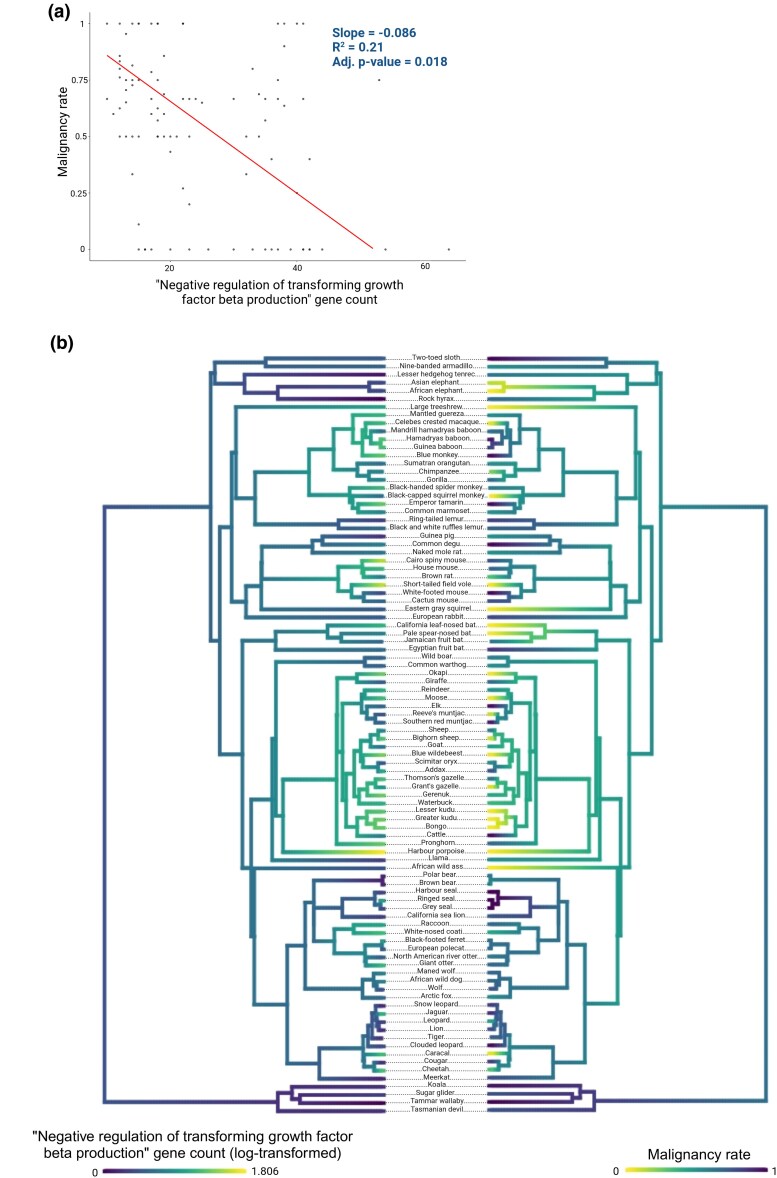
a) PGLS regression between the aggregate copy number of genes in set “negative regulation of transforming growth factor beta production” and rate of malignancy across mammals. b) Ancestral trait reconstruction of the aggregate gene count for the gene set “negative regulation of transforming growth factor beta production” (log transformed) and malignancy rate. Color scale shows the estimated evolutionary history of each trait, inferred using contMap function in the R package PhyTools (v2.1-1) (Revell 2012).

The gene set with the strongest evidence for association with malignancy rate was the gene ontology biological process “negative regulation of transforming growth factor beta production” (NR_TGFβ; *Fig. 2*). This gene set consisted of 12 genes (Table 1), and although, individually, none of these genes showed a statistically significant association between their copy number and malignancy rate, 11 genes exhibit the same directionality in their association, such that the aggregate count of genes within the set is significantly correlated with the malignancy rate across the phylogeny (Fig. 3).

**Table 1:** PGLS results for association between the copy number of individual genes within the negative regulation of TGFβ production gene set and malignancy rate

Gene	Estimate (coefficient)	Standard error	*T*-value	*P*-value	Adjusted *P*-value
*Cd24a*	−0.046	0.029	−1.5	0.11	0.55
*Cd2ap*	−0.067	0.032	−2.0	0.039	0.41
*Cdh3*	−0.058	0.036	−1.6	0.11	0.54
*Fbln1*	−0.082	0.035	−2.3	0.021	0.36
*Fn1*	−0.011	0.0049	−2.2	0.025	0.36
*Furin*	−0.047	0.015	−3.0	0.0034	0.11
*Gata6*	−0.033	0.014	−2.2	0.024	0.21
*Il13*	0.037	0.041	0.89	0.37	0.77
*Laptm4b*	−0.015	0.043	−0.36	0.71	0.97
*Met*	−0.091	0.025	−3.6	0.00041	0.12
*Tsku*	−0.049	0.013	−3.7	0.0003	0.063
*Tyrobp*	−0.097	0.041	−2.3	0.019	0.36

Genes were annotated using the house mouse (*M. musculus*) genome annotation.

**Fig. 4.**
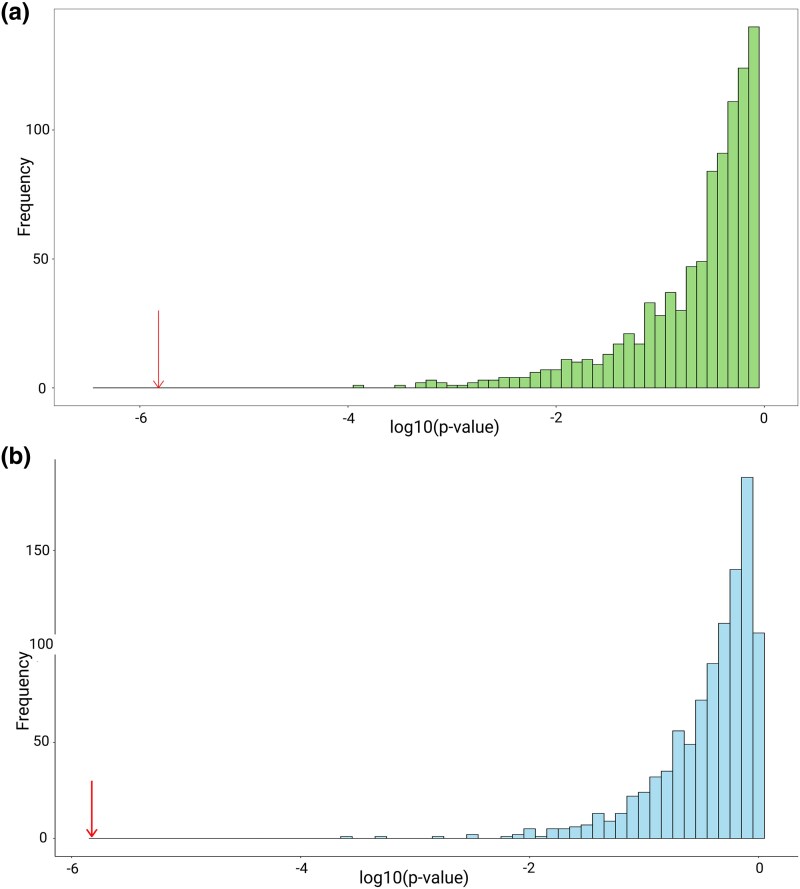
a) Distribution of *P*-values (log10 transformed) from a randomized test of PGLS models for association between aggregate gene count and malignancy rate. Simulated gene sets replicated the gene set “negative regulation of transforming growth factor beta production” by randomly sampling 12 genes with similar variance to those in the set. The arrow shows the actual *P*-value of the PGLS model for this set. b) Distribution of *P*-values (log10 transformed) from 1,000 iterations of a simulation test of PGLS between gene count of set “negative regulation of transforming growth factor beta production” and a random continuous phenotype simulated under Brownian motion. The arrow shows the actual *P*-value of the PGLS model with malignancy rate as the phenotype.

To assess the robustness of the observed associations between aggregate gene count and phenotype, two simulation strategies were employed (see the “Materials and Methods” section for details). In the first simulation strategy, random gene sets were sampled with the variance of the copy number of the genes matched to that of the genes in the gene set of interest. We then used PGLS to test for an association of aggregate copy number and malignancy. The *P*-value of the association between aggregate gene count for the NR_TGFβ gene set and malignancy rate was lower than for any of the simulated datasets (Fig. 4a), suggesting that the result is robust to the assumptions of the PGLS method. In a second simulation strategy, we simulated a random continuous phenotype, keeping the gene copy numbers for the NR_TGFβ gene set fixed at their observed values. Again, the *P*-values for the association of malignancy rate and the aggregate gene copy number of the NR _TGFβ set were lower than the *P*-value obtained for any of the 1,000 simulated phenotypes (Fig. 4b).

As an alternative test, we also treated the gene copy number as the response variable, with the malignancy rate as the explanatory variable. Given that the gene copy numbers are counts, we used phylogenetic generalized linear models (PGLMs) for this analysis, with a Poisson error structure. All 10 gene sets identified as significant in the original PGLS analysis retained their associations with malignancy rate (*P*-value < 0.05) in this analysis (supplementary table S2, Supplementary Material online).

### Gene Set Enrichment in Mammalian Life History Traits

**Fig. 5.**
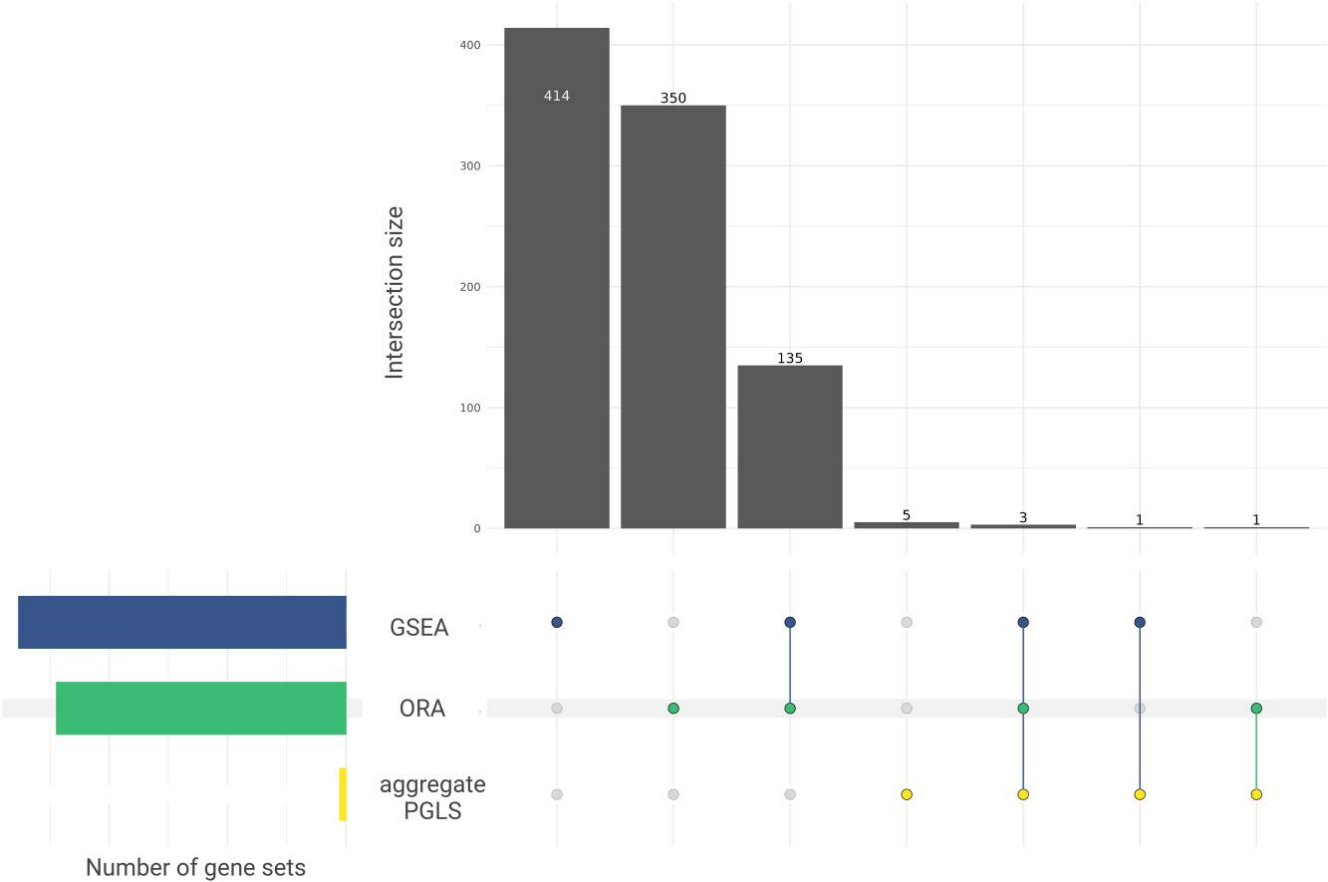
Upset plot of the gene sets associated with malignancy rate (adjusted *P*-value <0.05) across all three methods of gene set analysis.

In addition to the aggregate gene count PGLS analysis, we also applied standard gene set analysis methods to explore the association between copy number in gene sets and life history traits. Using the GSEA method, we found that all traits were significantly enriched for association with immune-related gene sets; however, the specific sets differed between traits (supplementary data S4, Supplementary Material online). Interestingly, enrichment for copy number associations with longevity and body size included several sets related to DNA repair (adjusted *P*-value <0.05), such as “DNA double strand break repair,” “G2M DNA damage checkpoint,” and “DNA double strand break response.” Of the ten gene sets associated with rate of malignancy in the aggregate gene count PGLS, four were also enriched for copy number association using GSEA (adjusted *P*-value <0.05; Fig. 5). These sets were gene ontology terms: “negative regulation of transforming growth factor beta production,” “negative regulation of transforming growth factor beta1 production,” “MHC class I peptide loading complex,” and “TAP complex binding.”

We also conducted an ORA to test if a higher proportion of genes from any gene set showed a nominally significant (uncorrected *P*-value <0.05) association with the life history traits (supplementary data S5, Supplementary Material online). Consistent with the GSEA results, several immune-related gene sets were enriched for association between copy number and life history traits (supplementary fig. S3, Supplementary Material online). Among the gene sets that were significantly enriched for an association with malignancy rate in ORA, 135 were also enriched in GSEA (Fig. 5), including sets such as “Ras protein signal transduction” and “Wnt signaling pathway.” Three gene sets with an association between aggregate gene count and malignancy rate (adjusted *P*-value <0.05) were also enriched in both GSEA and ORA. These three gene sets were “negative regulation of transforming growth factor beta production,” “MHC class I peptide loading complex,” and “TAP binding complex.”

## Discussion

Previous studies have suggested that the duplication of tumor suppressor genes could contribute to decoupling cancer risk from body size and extended longevity (Abegglen et al. 2015; Caulin et al. 2015; Sulak et al. 2016). However, to the best of our knowledge, these studies have not extended beyond genes annotated as tumor suppressor genes or oncogenes. In this study, we have performed a comprehensive survey of gene copy number of all protein-coding genes in 94 mammals. We found the copy number of 15 orthogroups to be associated with malignancy rate; however, no orthogroups were significantly associated with longevity, body size, neoplasia prevalence, or malignancy prevalence following correction for multiple testing, using an FDR threshold of 0.05. We also identified gene sets for which changes in the aggregate gene count were associated with rate of malignancy across the mammalian phylogeny.

Among the 15 orthogroups associated with malignancy rate, the strongest association was for *nuclear receptor coactivator 4* (*Ncoa4*). This gene encodes an androgen receptor coactivator and is involved in the process of ferroptosis and ferritinophagy (Gryzik et al. 2021). Both ferritinophagy and ferroptosis play significant roles in the regulation, initiation, and progression of cancer; due to the abnormal iron metabolism in cancer cells, ferroptosis can act as a tumor suppression mechanism (Wang et al. 2023). Other genes of interest among these 15 include *Tusc3*, a candidate tumor suppressor gene (Feng et al. 2018; Sun et al. 2022a), and *Nek8*, which plays a role in cell cycle progression and has been reported to affect cancer progression (Cao et al. 2023).

The lack of an association between the copy number of any individual gene and longevity or body size contrasts with previous studies that have found such associations (Sulak et al. 2016; Tollis et al. 2020; Vazquez and Lynch 2021). For example, the copy number of tumor suppressor gene *TP53* has a reported association with body size and longevity in the elephant lineage (Caulin and Maley 2011; Abegglen et al. 2015; Caulin et al. 2015; Vazquez and Lynch 2021). However, these studies included pseudogenes and retrogenes in the copy number count, whereas here we restricted to protein-coding genes. Here, we also look for association across a wider range of taxa, suggesting that gene copy number is not associated with longevity or body size in the taxa investigated in this study.

The cancer prevalence data analyzed here were from the study by Compton et al. (2024), in which cancer prevalence was estimated from the necropsies of zoo animals. These data have some important limitations for our purposes; namely, cancer prevalence estimates were based on tens of individuals. These small sample sizes may lead to increased error and bias; therefore, results should be interpreted with caution. Another potential problem in our analysis is reliance on annotated genomes for discovery of orthogroups. Our ability to identify orthogroups was limited by the quality of genome annotations. We have addressed this issue by evaluating the annotations with BUSCO and removing species with a low completeness score. We also used the total number of protein-coding genes in the species as a covariate in our analysis to account for potential bias from variation in proteome size between species.

In addition to investigating the association between gene copy number and life history traits, we also investigated the association between traits and the aggregate copy number in gene sets. Applying gene set analysis to gene copy number data allowed us to identify processes and pathways for which the gene copy number aggregated across all genes in the set is associated with longevity, body size, or cancer occurrence in mammals. Using PGLS, we found that the gene count of 10 gene sets was associated with malignancy rate. Eight of these cases had a negative relationship with rate of malignancy across species, with expansion of these gene sets associated with lower rates of malignancy. These included gene sets related to transforming growth factor (TGF) and various immune processes such as transporter associated with antigen processing (TAP) binding, the major histocompatibility complex (MHC), and natural killer cell activation. The remaining two sets had a positive relationship with rate of malignancy, such that expansion of these sets was associated with increased rates of malignancy. The gene set with the strongest statistical evidence of association was NR_TGFβ. This gene set was the only one to survive family-wise correction for multiple testing (using the Bonferroni and Hommel methods and corrected *P*-value <0.05) from the aggregate PGLS analysis and was one of three sets that were also enriched in both GSEA and ORA analyses. TGF beta (TGF-β) is part of the TGF-β signaling pathway and is highly expressed in a variety of tumors, such as bladder cancer (Zhang et al. 2016), ovarian cancer (Wang et al. 2012), and hepatocellular carcinoma (Peng et al. 2017). At the onset of malignancy, TGF-β acts as a tumor suppressor. It plays a role in the removal of malignant cells by reducing cell proliferation and differentiation, triggering apoptosis (Baba et al. 2022). TGF-β also promotes the activation of tumor suppressor genes (Katz et al. 2013). Our results suggest that the increases in copy number of genes in the TGF-β pathway may reduce malignancy rate in mammals.

To confirm the robustness of the result for this gene set, we performed two simulation tests. In the first test, we generated 1,000 simulated gene sets by randomly sampling genes that matched the variance in copy number of the NR_TGFβ set and then ran PGLS for these simulated sets for association with malignancy rate. The observed *P*-value was smaller than the *P*-values obtained in any of the simulated data sets. However, due to the large number of gene sets tested in this study, it is impractical to simulate the entire dataset to rule out chance findings completely. In the second simulation test, we used the observed copy numbers of genes in the NR_TGFβ set and simulated a random continuous phenotype, under a Brownian motion model. This simulation was performed to evaluate whether the use of the observed gene counts as an explanatory variable could result in a false-positive association between gene copy number and a randomly evolving continuous phenotype. In 1,000 simulation runs, the *P*-values for the simulated phenotypes were consistently larger than the *P*-value observed in the original analysis.

The NR_TGFβ association was also found by both GSEA and ORA analyses. The association between NR_TGFβ and malignancy rate was, therefore, supported both by a self-contained test (PGLS applied to the aggregate gene count in the gene set) and by multiple competitive tests (GSEA, ORA, and our randomization test) that compare the gene set against background genes. There were 135 gene sets that overlapped between the GSEA and ORA results, with enrichment for rate of malignancy (Fig. 5). Interestingly, sets that were enriched in both analyses included sets associated with Wnt and Ras signaling, both of which are major pathways involved in cancers (Jeong, Ro and Choi 2018). Three gene sets enriched in both GSEA and ORA were also found to be significantly associated with malignancy rate in the aggregate count PGLS. These sets include NR_TGFβ, as previously discussed, and the ontology terms “TAP complex binding” and “MHC class I peptide loading complex.” Both the TAP complex and the MHC play an integral role in the immune system and are involved in antigen presentation. Abnormalities in both TAP and MHC class I molecules impair the immune system's ability to detect and control malignancies (Lankat-Buttgereit and Tampé 2002; Dhatchinamoorthy et al. 2021). The results presented here suggest that an increase of copy number of genes involved in these processes is associated with a decrease in malignancy rate across mammals.

We found no gene sets to be associated with body size or lifespan using PGLS applied to the aggregate gene count in the gene set. However, associations between gene sets and both of these traits were identified using GSEA and ORA methods. There are a number of caveats to these methods, however, that limit our confidence in these results. For example, competitive tests, such as GSEA, are sensitive to choice of ranking metric (Zyla et al. 2017). Here, we make use of the *P*-values from our individual gene PGLS, which are sensitive to the variance in copy number across the phylogeny. For example, genes with highly variable copy numbers may dominate the top of the ranked list. This can lead to false positives or inflated significance for gene sets that have inherent variability rather than an association with the trait of interest. Sets with little variation in copy number across the tree cannot have strong association to phenotypes and will therefore be absent from the upper ranks of the list. In the case of ORA, gene sets with highly variable genes are more likely to contain genes that meet the significance threshold, favoring larger, more variable sets. Indeed, genes associated with highly variable copy number, such as immune genes, featured strongly among our enriched gene sets. The prominence of immune-related gene sets in both GSEA and ORA results is consistent with previous studies that have found the evolution of immunity-related gene sets to be correlated with body size (Huang et al. 2021; Sun et al. 2022b); however, given the substantial limitations of these methods, the results from these analyses should be interpreted with caution. The novel method proposed in this study, which involved applying PGLS to the aggregate gene count in the gene set, provides a self-contained test that is not affected by this bias.

In conclusion, this study provides a more comprehensive picture of processes and pathways in which gene copy number is associated with key life history traits, potentially helping to shed light on long-standing questions relating to the evolution of cancer prevalence across mammals. While we did not find strong evidence that supports the hypothesis that gene copy number plays a role in decoupling cancer risk from changes in longevity and body size, we have identified several sets of genes for which gene copy number is correlated with malignancy rate across mammals. Notably, the expansion of gene sets related to the TGF-β pathway was associated with decreased malignancy rate, which is consistent with the tumor suppressive role of the TGF-β in the early stages of cancer progression. Overall, these results suggest that cancer risk in mammals can be modified in part by gene copy number changes and highlight pathways and processes in which changes in copy number may contribute to variation in cancer risk across mammals.

## Materials and Methods

### Data Collection

We obtained publicly available genomes for 105 mammals (NCBI [Sayers et al. 2022], Ensembl [Martin et al. 2023], and UCSC [Kent et al. 2002]) (supplementary data S1, Supplementary Material online), spanning 16 different orders (Afrosoricida, Artiodactyla, Carnivora, Cetacea, Chiroptera, Cingulata, Dasyuromorphia, Diprotodontia, Hyracoidea, Lagomorpha, Perissodactyla, Pilosa, Primates, Proboscidea, Rodentia, and Scandentia). An ultrametric phylogenetic tree of these species was inferred using timetree.org (Kumar et al. 2022) (Fig. 1a). Of these genomes, protein sequences were available and downloaded for 54 species (Blin 2023). The remaining genomes were annotated using liftoff (Shumate and Salzberg 2021), using a reference species genome and annotation. To ensure the most accurate lifted annotation, we selected the closest relative, for which we had assembly and annotation, as reference. Proteomes were subsequently created from the annotations using GFFtK (v23.11.2) (Palmer 2023). Proteome completeness was assessed using BUSCO (Simão et al. 2015), and 11 species with a completeness of <50% were discarded from further analysis (Fig. 1a).

Phenotypic data were collected from a previous study of cancer across vertebrates (Compton et al. 2024). In the study, necropsy records were obtained from zoological institutions, aquariums, and other facilities that house animals under managed care. Of cases where neoplasia was observed, malignant and benign neoplasms were distinguished based on the diagnoses in necropsy reports written by board-certified pathologists. In this study, we analyze three cancer phenotypes: prevalence of neoplasia, prevalence of malignancy, and malignancy rate. The malignancy rate was determined by calculating the proportion of neoplasia identified as malignant. Body size and longevity data were obtained from PanTHERIA (Jones et al. 2009) (Fig. 1).

### Identification of Orthogroups

Gene orthogroups were inferred using OrthoFinder (v2.5.5) (Emms and Kelly 2019), to estimate the gene copy number for all protein-coding genes. In the annotation of orthogroups, we utilized the house mouse (*Mus musculus*) genes within each orthogroup as a reference, selecting mouse as the basis due to its superior genome annotation and resources compared with other nonmodel species included in our study. This approach allowed for reliable identification of constituent genes within each orthogroup.

### PGLS Analysis of Gene Copy Number and Life History Traits

To test for association between gene copy number and phenotypes, we used PGLS to control for nonindependence of phenotypes in related species. In the PGLS model, the phenotypes played the role of the response variable, while gene copy number was the predictor, allowing us to assess the influence of gene count on the traits of interest. For life history traits (body size and longevity), PGLS was carried out using the R package phylolm (v2.6.2) (Tung Ho and Ané 2014). We employed the lambda (λ) method to estimate phylogenetic signal and account for the degree of phylogenetic dependence in the model. In each model, the other phenotype was included as a covariate (for example, in the model for body size, longevity was included as a covariate). For associations with cancer phenotypes, species datapoints were weighted by the square root of the number of necropsies, to address the variation in sample numbers and to limit noise from estimates based on few individuals (Revell 2012; Compton et al. 2024). In all PGLS models, the number of protein sequences present in a species proteome was used as a covariate, to account for biases arising from the variation in proteome size. To ensure the reliability of our analysis, we restricted PGLS to orthogroups where more than 50% of species had a nonzero copy number, as we could not definitively determine if zero values were true absences or artifacts of poor annotation. We controlled for multiple testing by using the Benjamini–Hochberg method (Benjamini and Hochberg 1995) with an FDR of 5%.

A further PGLS analysis was conducted to test for associations between the aggregate gene count of gene sets and the phenotypes. To collect aggregate gene counts, orthogroups were grouped into gene sets as defined by the Molecular Signature Database (mouse) hallmark gene sets, curated gene sets of canonical pathways, and ontology gene sets (Mootha et al. 2003; Subramanian et al. 2005). In addition to investigating the total copy number of genes within predefined gene sets, we also aggregated orthogroups corresponding to tumor suppressor genes and oncogenes, as described by COSMIC (accessed November 2023) (Sondka et al. 2018). The total number of genes found within each set was calculated for each species, including all protein-coding copies. PGLS analysis was carried out on the aggregate counts as described above. However, instead of using the total number of protein sequences present in a species’ proteome as a covariate, we used the number of proteins found in orthogroups containing a mouse gene. This adjustment was necessary because the gene sets were defined by mouse genes, meaning only sequences corresponding to a mouse gene could be utilized. Based on the results of this analysis, we plotted the malignancy rate and gene set size for the gene set “negative regulation of transforming growth factor beta production” by reconstructing ancestral states at internal nodes using maximum likelihood with contMap from the R package phytools (v2.1-1) (Revell 2012).

We carried out a further two simulation tests to assess the robustness of our PGLS results. In the first simulation test, given a set of interest, we produced 1,000 replicates of the set by randomly selecting the same number of genes with a similar variance to those found within the original set. The PGLS analysis was then carried out on these random gene sets, and the test statistics were compared with the original results. In a second simulation strategy, we retained the observed gene copy numbers for the gene set of interest and simulated a random continuous phenotype under a Brownian motion model. A PGLS was then performed for 1,000 iterations of this simulation, testing the association between gene copy number and the simulated phenotypes. The test statistics from these simulations were compared with the original PGLS results for the gene set of interest to evaluate the likelihood of obtaining a false-positive association by chance.

As an alternative to PGLS, we fitted PGLMs to the gene sets that showed a significant association (adjusted *P*-value <0.05) with malignancy rate. In this analysis, we reversed the roles of gene count and trait, treating gene count as the response variable and malignancy rate as the predictor. We used a Poisson error structure to model the gene copy number counts.

### Gene Set Analysis

In addition to the application of PGLS and phylogenetic generalised linear mixed (PGLM) model methods to the aggregate gene count in gene sets, we also applied standard gene set analysis methods to identify processes and pathways that show evidence for enrichment for genes with an association between their copy number and longevity, body size, or cancer prevalence. Results from the individual gene PGLS analysis were used to conduct a GSEA. For each phenotype, genes were separated into two groups, dependent on whether its copy number had a positive or negative correlation with the phenotype. The genes were then ranked by *P*-value. GSEAPreranked (Mootha et al. 2003; Subramanian et al. 2005) was used to identify gene sets overrepresented at the top and bottom of the ranked list. Gene sets positively enriched for the smallest *P*-values were indicative of an association between gene copy number and the given phenotype. A classic (nonweighted) enrichment statistic was used with default parameters. Gene sets utilized for this analysis were the mouse hallmark gene sets, curated gene sets of canonical pathways, and ontology gene sets (Liberzon et al. 2011; Castanza et al. 2023).

We also conducted a ORA, to identify processes and pathways that were significantly enriched among the genes with a nominal *P*-value < 0.05 for each trait. Enrichment was carried out using the clusterProfiler package (v4.8.3) (Wu et al. 2021) for ontology gene sets (The Gene Ontology Consortium 2019) and pathways from the Kyoto Encyclopedia of Genes and Genomes (KEGG) database (Kanehisa et al. 2023). A significance threshold of FDR-corrected *P*-values <0.05 was applied to the ORA results.

## Supplementary Material

msaf056_Supplementary_Data

## Data Availability

This study used publicly available genomes documented in the Supplementary material. Results generated from this study are included in the manuscript and Supplementary material. The codes used to generate the results presented in this study are available from our GitHub (https://github.com/sophie-03/gene_cn_lifehistory).
