# Supplementary material for: Variable Gene Copy Number in Cancer-Related Pathways Is Associated With Cancer Prevalence Across Mammals
Source: Mol Biol Evol. 2025 Mar 20;42(3):msaf056. doi: 10.1093/molbev/msaf056 (PMC11954591; doi:10.1093/molbev/msaf056)
Supplement: msaf056_Supplementary_Data [file msaf056_supplementary_data.zip › supplementary_figures-and_tables_revised.pdf]

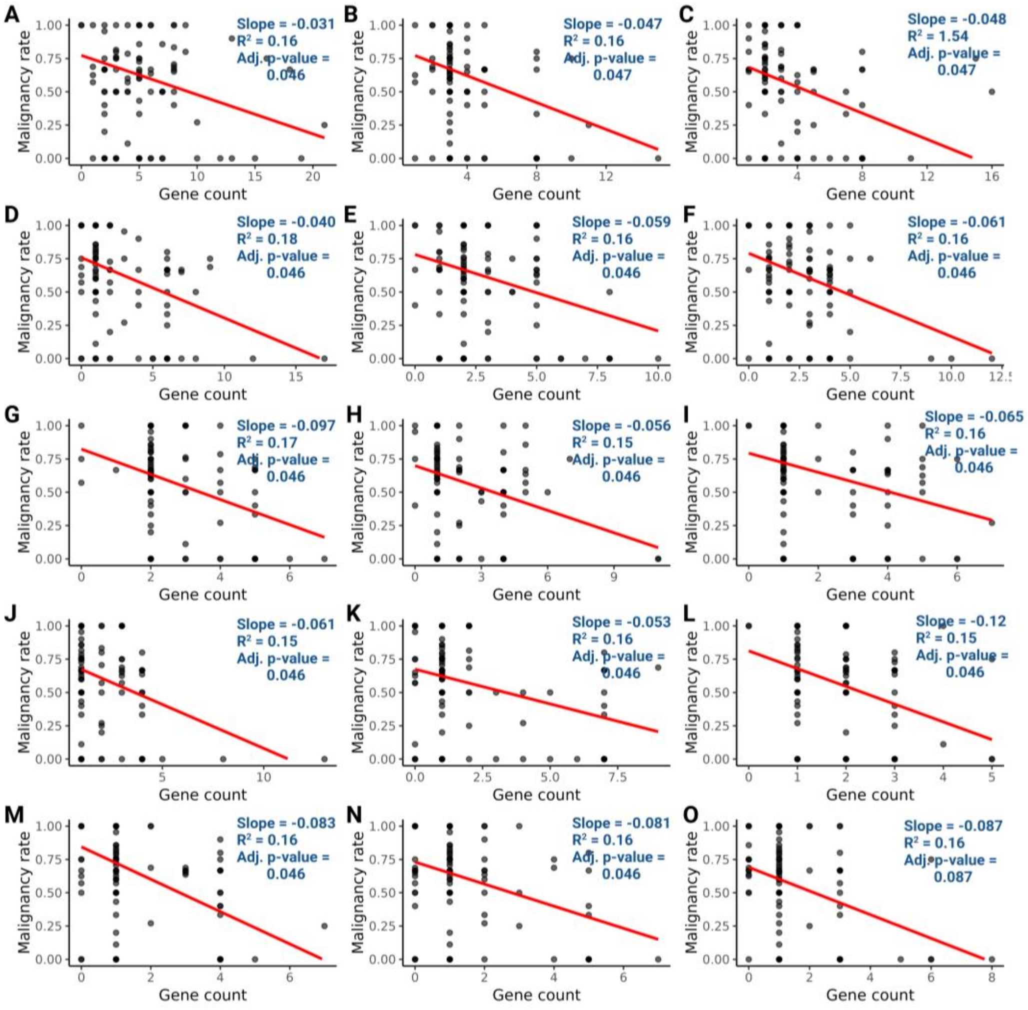

Supplementary Figure 1. PGLS regression between malignancy rate and the aggregate copy number of genes in orthogroups **(A)** OG0000590, **(B)** OG0001296, **(C)** OG0001584, **(D)** OG0002495, **(E)** OG0002684, **(F)** OG0002860, **(E)** OG0002890, **(G)** OG0003814, **(H)** OG0004404, **(I)** OG0004572, **(J)** OG0004601, **(K)** OG0005697, **(L)** OG0006344, **(M)** OG0007672, **(N)** OG0008278. The genes comprising each orthogroup are listed in Supplementary Table 1.

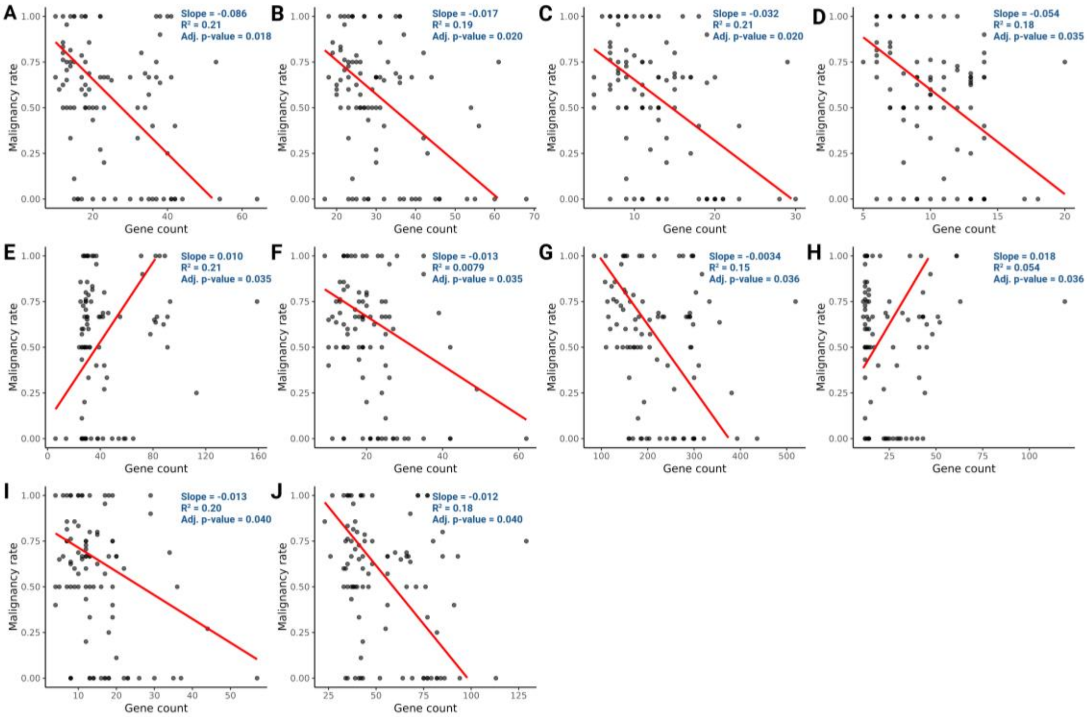

Supplementary Figure 2. PGLS regression between malignancy rate and the aggregate copy number of genes in gene sets: **(A)** Negative regulation of transforming growth factor beta production, **(B)** Transforming growth factor beta-1 production, **(C)** Negative regulation of transforming growth factor beta-1 production, **(D)** Positive regulation of ER-associated ubiquitin-dependant protein catabolic process, **(E)** Axonemal dynein complex, **(F)** MHC class I peptide loading complex, **(G)** Natural killer cell activation, **(H)** Regulation of neuromuscular junction development, **(I)** TAP complex binding, **(J)** Cell migration involved in heart development.

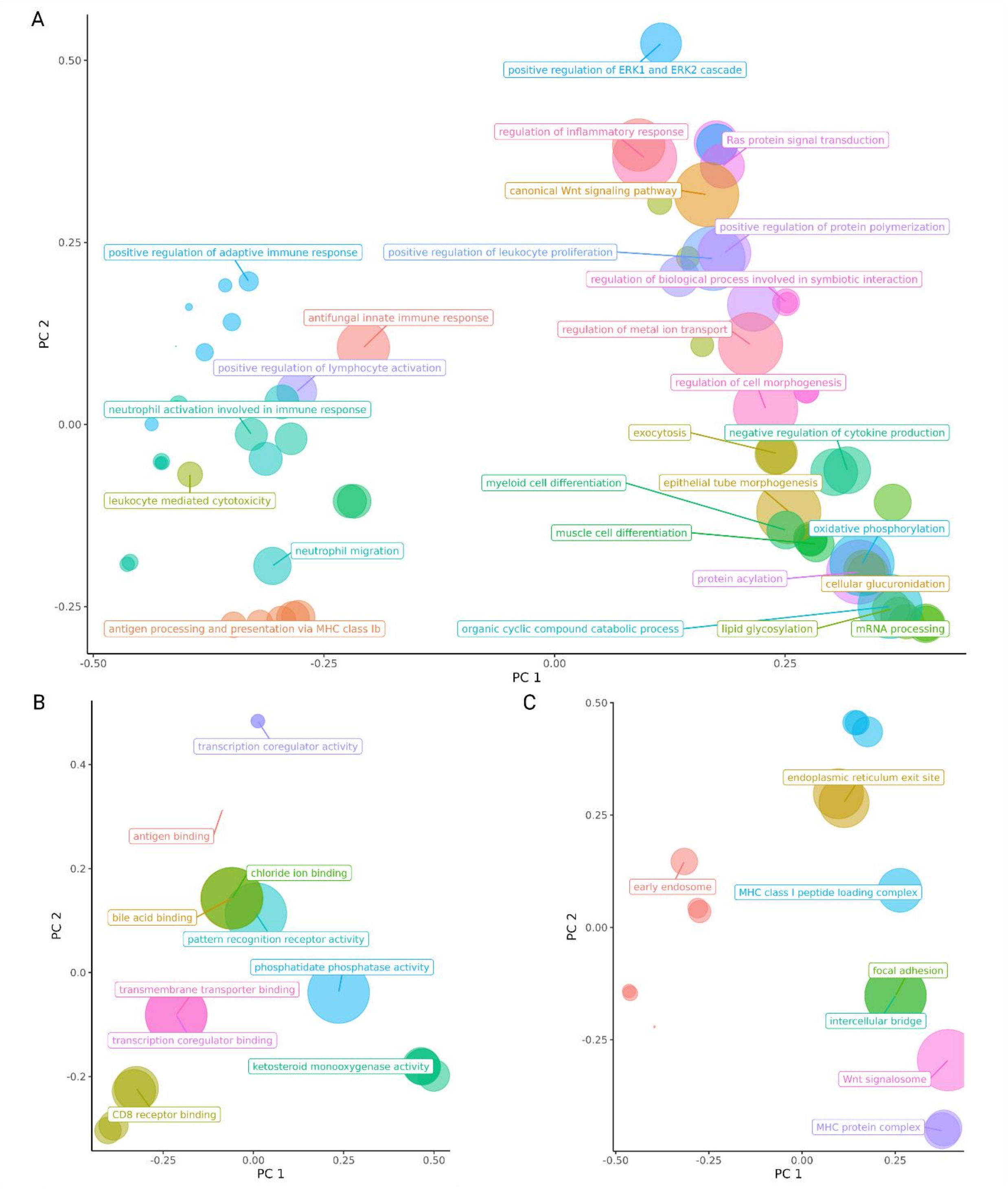

Supplementary Figure 3. Scatter plot represents Gene Ontology (GO) representative terms that are significantly enriched in both GSEA and ORA analysis for association between copy number and malignancy rate, for GO categories (**A**) biological process, (**B**) molecular function and (**C**) cellular component. Distance between points represents the similarity between terms, and the axes are the first two components of applying PCoA to the (di)similarity matrix. Size of the point represents the number of genes the GO term contains.

Supplementary Table 1. Orthogroups with an association between copy number and malignancy rate (FDR adjusted p-value < 0.05). Genes within orthogroups were annotated using the house mouse (*Mus musculus*) genome annotation.

Orthogroup	Estimate (coefficient)	Standard error	T-value	P-value	Adjusted P-value	Genes
OG0000590	-0.031	0.0074	-4.2	6.0e-05	0.046	*A3galt2, Glt6d1, 4930402F06Rik, Ggta1, 4930568D16Rik*
OG0001296	-0.047	0.011	-4.0	9.3e-05	0.047	*Kctd5, Kctd2, Kctd17*
OG0001584	-0.048	0.011	-4.0	1.0e-04	0.047	*Tusc3, Magt1*
OG0002495	-0.040	0.0090	-4.5	1.6e-05	0.046	*Ncoa4*
OG0002684	-0.059	0.014	-4.1	6.5e-05	0.046	*Cnbp, Zcchc13*
OG0002860	-0.061	0.014	-4.1	7.0e-05	0.046	*Hmgb3*
OG0002890	-0.097	0.022	-4.3	3.9e-05	0.046	*Ednrb, Ednra*
OG0003814	-0.056	0.013	-4.1	8.0e-05	0.046	*Gfod2*
OG0004404	-0.065	0.015	-4.2	5.4e-05	0.046	*Stmn4*
OG0004572	-0.061	0.014	-4.1	8.4e-05	0.046	*Flot1*

OG0004601	-0.053	0.012	-4.2	5.9e-05	0.046	*Zfp114, Zfp235, Zfp114, Zfp108, Zfp109, Zfp111*
OG0005697	-0.12	0.029	-4.1	7.2e-05	0.046	*Prr15l*
OG0006344	-0.083	0.019	-4.2	5.0e-05	0.046	*Nek8*
OG0007672	-0.081	0.019	-4.1	6.5e-05	0.046	*B3galnt2*
OG0008278	-0.087	0.020	-4.1	6.5e-05	0.046	*Tmem80*

Supplementary Table 2. Results of PGLMs for gene sets that have an association with malignancy rate.

Gene set	Gene ontology category	Estimate (coefficient)	Intercept	Standard Error	Z-value	P-value
Negative regulation of transforming growth factor beta production	Biological process	-0.086	2.9	0.036	-2.3	0.018

Transforming growth factor beta1 production	Biological process	-0.16	3.0	0.021	-7.6	2.5e-14
Negative regulation of transforming growth factor beta1 production	Biological process	-0.17	2.1	0.031	-5.7	9.3e-09
Positive regulation of ER associated ubiquitin dependent protein catabolic process	Biological process	-0.21	1.9	0.022	-9.5	1.8e-21
Axonemal dynein complex	Cellular component	0.12	3.0	0.030	3.9	8.0e-05
MHC class I peptide loading complex	Cellular component	-0.21	3.14	0.042	-5.1	3.2e-07

Natural killer cell activation	Biological process	-0.080	4.8	0.020	-3.9	8.4e-05
Regulation of neuromuscular junction development	Biological process	0.13	2.6	0.043	3.1	0.0014
TAP complex binding	Molecular function	-0.32	2.7	0.067	-4.8	1.4e-06
Cell migration involved in heart development	Biological process	-0.14	3.5	0.020	-7.1	7.6e-13
